# Three Method-Combination Protocol for Improving Purity of Extracellular Vesicles

**DOI:** 10.3390/ijms21093071

**Published:** 2020-04-27

**Authors:** Thomas Simon, Anish Kumaran, Diana-Florentina Veselu, Georgios Giamas

**Affiliations:** Department of Biochemistry and Biomedicine, School of Life Sciences, University of Sussex, Brighton BN1 9QG, UK; anish.kumaran26@gmail.com (A.K.); dv80@sussex.ac.uk (D.-F.V.)

**Keywords:** extracellular vesicles, size exclusion chromatography, differential ultracentrifugation

## Abstract

Extracellular vesicles (EVs) are nanosized structures able to carry proteins, lipids and genetic material from one cell to another with critical implications in intercellular communication mechanisms. Even though the rapidly growing EVs research field has sparked great interest in the last 20 years, many biological and technical aspects still remain challenging. One of the main issues that the field is facing is the absence of consensus regarding methods for EVs concentration from biofluids and tissue culture medium. Yet, not only can classic methods be time consuming, commercialized kits are also often quite expensive, especially when research requires analyzing numerous samples or concentrating EVs from large sample volumes. In addition, EV concentration often results in either low final yield or significant contamination of the vesicle sample with proteins and protein complexes of similar densities and sizes. Eventually, low vesicle yields highly limit any further application and data reproducibility while contamination greatly impacts extensive functional studies. Hence, there is a need for accessible and sustainable methods for improved vesicle concentration as this is a critical step in any EVs-related research study. In this brief report, we describe a novel combination of three well-known methods in order to obtain moderate-to-high yields of EVs with reduced protein contamination. We believe that such methods could be of high benefits for in vitro and in vivo functional studies.

## 1. Introduction

Even though extracellular vesicles (EVs) have been described as ‘useless cell debris’ for decades, they have been recognized lately as key constituents of inter-cellular communication pathways [1,2]. EVs are lipid bilayer membrane-enclosed particles that are naturally released from cells [3]. Such cargo vessels transfer lipids, proteins, various fragments of nucleic acids and metabolic components to adjacent cells or to distant sites in the body, mainly through the circulatory system. For these reasons, EVs have been reported to play central roles in both normal and pathological conditions, such as pregnancy and cancer [1,4,5]. Similarly, EVs also play a unique role in spreading various pathogens like viruses and prions from one cell to another [6].

EVs can be classified into two clearly defined subtypes based on their sizes, namely “small EVs” (sEVs) with a size between 50–200 nm, or “medium/large EVs” (m/l EVs) with a size range between 200 nm–1 µm in diameter. This nomenclature is now preferred to the classic, yet quite vague, terms “exosomes”, “microvesicles” or “oncosomes”, as high heterogeneity in terms of size, marker expression and origins have been reported for each subpopulation, leading to overlaps between them [2]. 

Interest in the EVs has significantly grown in the medical research community over the past decade. Indeed, a thorough and comprehensive description of such EVs-dependent pathways may provide new inputs to develop effective treatments [5,7]. Mostly focusing on the sEVs subtype, the field has failed so far to establish essential technical standards, such as an optimal sEVs concentration/isolation method [1,2]. Thus, there is no consensus regarding that important matter whatsoever, raising important concerns regarding data reliance and reproducibility. As a matter of fact, currently available methods for sEVs concentration can hardly provide both high yield and high purity at once [8,9]. Consequently, such lack of effective techniques directly affects biomarker discovery and functional studies for which description of exclusively sEVs-related mechanisms and cargo is needed. 

EVs are most commonly separated and concentrated from cell culture conditioned medium or human biofluids by differential ultracentrifugation (UC). This method allows for the separation of small particles, such as m/l and sEVs, from other larger ones, such as cell debris, based on their respective density and size, through successive increases of centrifugation forces and time. Differential UC is easy to perform, moderately time-consuming and does not require much technical expertise. Nonetheless, even when the parameters are optimized, the process results in a mixture of EVs concentrated along with particles of the same buoyant density and size range. In other words, large proteins and protein aggregates contaminate the EVs preparation. The co-isolated non-EVs structures are most often lipoproteins such as APOA1/2 or APOB, and Albumin [10]. Alternatively, researchers use various different methods such as size exclusion chromatography (SEC), filtration, precipitation, density gradients or immuno-isolation [2]. Yet, such protocols are not perfect as the final yield is often low and the purity is not optimal. In addition, these methods are usually commercialized in the form of expensive kits, altogether making them hardly applicable to extensive in vitro functional studies. For all these reasons, combining some of these methods seems to be the only sensitive strategy to substantially improve both the purity and the concentration of the final EVs preparation [2,10]. In theory, SEC makes it possible to separate the EVs from other particles, mainly proteins complexes and lipoproteins based on their size, through running a sample on a column made of resin with a define pore size. Consequently, such methods should help purify EVs samples obtained through UC [11,12]. 

For all these reasons, the present study has been undertaken in an attempt to improve the purity of sEVs preparation with the extra goal to maintain an important final concentration so that extensive functional studies are feasible. To do so, we have: (1) concentrated putative sEVs through UC, followed by (2) SEC in order to exclude protein contaminants from the assumed sEVs preparation. Finally, (3) an extra step was performed post-SEC using a centrifugal filter device in order to improve the concentration of the final sEVs samples. The final concentrated sEVs are therefore less contaminated as compare to sEVs separated using UC. Moreover, although there was a marginal loss during the process, we observed that the structure and size of sEVs were intact following all these steps.

## 2. Results and Discussion

Despite a constant and rapid evolution of the techniques and methods in the EVs field, researchers are yet to reach a consensus regarding the particle concentration step that is critical for any EVs-focused study [13]. However, they largely agree on the higher performance of combinational protocols over single-method approaches, even though proper EVs isolation/purification still seems unrealistic. Indeed, obtaining high concentrations of EVs coupled to acceptable sample purity is still hardly feasible [1,2]. Yet, reaching such a goal would be of high value for current functional studies, as it would make it possible to perform reliable and reproducible experiments for deciphering EVs-specific mechanisms. Indeed, as mentioned in the latest update to the MISEV2018, highly purified EVs should be used when one wants to associate a function or marker expression to vesicles as compared with other potentially present particles [2]. 

**Figure 1:**
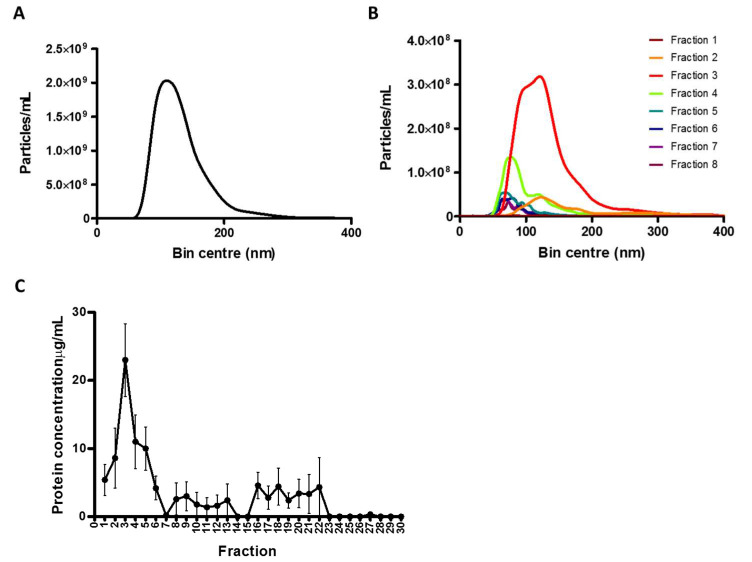
Nanoparticle tracking analysis and protein concentration measurement in initial ultracentrifugation sample and fractions from size exclusion chromatography (Step 1 and 2). Particle samples obtained following step 1 and 2 of the 3 method-combination protocol were processed to nanoparticle analysis (NTA) and protein concentration measurement. (**A**) NTA of the initial ultracentrifugation (UC) sample. Sample was diluted (1/50) in filtered sterile phosphate buffer solution (PBS) and measured using a Nanosight NS300. (**B**) NTA of the fractions from size exclusion chromatography (SEC). The initial UC sample was processed through SEC and fractions were measured by NTA. Fractions were diluted (1/20) in filtered PBS and measured using a Nanosight NS300. (**C**) Protein concentration measurement in the SEC fractions. Protein concentration was measured using a Nanodrop 200. The mean ± SEM of *n* = 5 independent experiments is shown.

In the present study, conditioned medium was collected from confluent glioblastoma (GBM) cells. Differential UC was then performed so that the original putative sEVs samples could be obtained. Following, nanoparticles analysis (NTA) was employed to determine the concentration of particles in the original samples (1.63 × 10^11^ particles/mL, Figure 1A). 

SEC was performed following this initial step. As shown in Figure 1B, NTA measurements revealed the presence of particles of the sEVs sizes mainly in SEC fractions 2, 3 and 4 (0.43, 2.70 and 0.74 × 10^10^ particles/mL, respectively). In addition, further Nanodrop analysis showed that SEC fractions 2, 3 and 4 (8.6, 23 and 11 µg/mL, respectively) showed the highest protein content, confirming the detection of putative EVs in the earliest fractions (detection of sEVs-associated proteins) and suggesting the presence of protein contaminants in the latest (Figure 1C). 

**Figure 2:**
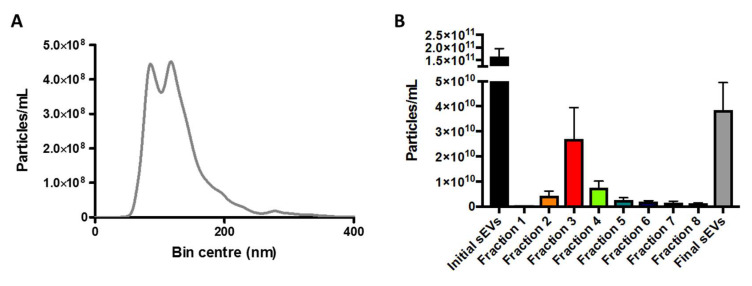
Comparison of particle concentration in final putative sEVs sample versus ultracentrifugation sample and fractions from size exclusion chromatography (Step 3). Final putative sEV sample was obtained following concentration of selected size exclusion chromatography (SEC) fractions using an Amicon Ultra 0.5 device – 30k. (**A**) Nanoparticle tracking analysis (NTA) of the final putative sEV sample. Sample was diluted (1/50) in filtered PBS and analyzed using a Nanosight NS300. (**B**) Particle concentration of the initial UC sample, fractions from SEC and final putative sEVs sample. The mean ± SEM of *n* = 5 independent experiments is shown.

Accordingly, fraction 3 was pooled with either fraction 2 or 4 and concentrated in a 100 µL of sterile phosphate buffer solution (PBSs). NTA of this final sample showed a particle concentration of 3.84 × 10^10^ particles/mL, which was 4.2× lower as compared to the original concentration obtained by UC (Figure 2A,B).

**Figure 3:**
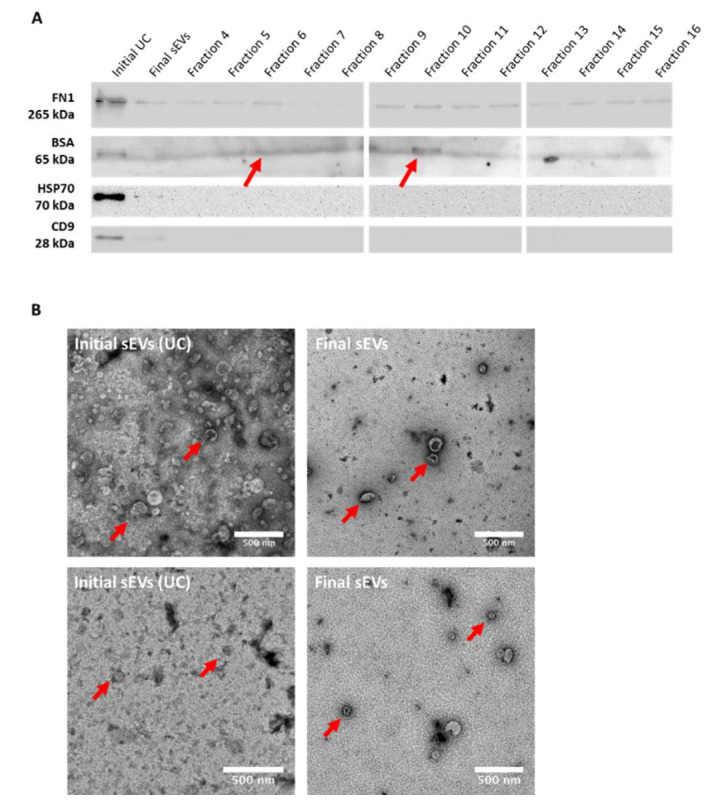
Validation of sEV concentration and decreased protein contamination. (**A**) Western blotting detection of fibronectin (FN1), bovine serum albumin (BSA), HSP70 and CD9 in initial (UC), final sEVs and SEC fractions. (**B**) TEM detection of sEVs (×20k magnification and zoom). Red arrows show sEVs. Representative pictures are shown. Scale bar = 500 µm.

Western blotting for sEVs markers, namely CD9 and HSP70, fibronectin (FN1) and described EVs sample contaminant albumin (BSA), was then performed. Data revealed exclusive expression of CD9 and HSP70 in both the original UC and final putative sEVs samples, validating the EVs concentration by both the UC method and the three method-combination protocol. The expression of EVs markers was lower in the final sEVs as compared to the UC sample (Figure 3A). BSA expression was mostly observed in the initial UC sample, fraction 5, fraction 6 and fraction 10. In addition, as shown in Figure 3A, a decrease of the FN1 expression was observed in the final sEVs sample as compared to the original UC sample. Yet, FN1 expression was also detected in all the SEC fractions with a slight decrease in fractions 7 and 8, and a slight increase in fractions 9 and 10. 

Finally, transmission electron microscopy (TEM) was performed in order to observe the structure/membrane integrity of the final sEVs sample as compare to the original UC one (Figure 3B). As seen in Figure 3B, final particles appear very similar structure wise as compare to original ones from the UC sample, displaying an apparently intact lipid bilayer membrane. Particle concentration appeared much lower in the final sample as compare to the original UC sample, confirming the NTA observations. Moreover, fewer debris and sEVs aggregates could be observed in TEM pictures of the final sEVs sample, as compared to the original UC one. 

Using our combination of methods, we observed that our final sEVs samples presented with fewer debris and particle aggregates as compared to our original samples obtained by UC. Altogether, it appears that our three method-combination protocol produced concentrated sEVs samples with enhanced purity as compared to the commonly used UC protocol. We can therefore confirm that combining UC and then SEC, in this order, allows: 1) to use large amounts of cell culture conditioned medium/biofluids for high sEVs concentrations and 2) to separate particles from protein contaminants found in the UC concentrated preparations. Final centrifugal concentration then allows for reducing sample dilution due to SEC. 

Our present method might be especially valuable for in vitro functional studies, as one of the strengths here is to make possible using very large volumes (>100 mL) of starting material. The final concentration of sEVs in this way is high enough to perform multiple validation and further functional/phenotypic experiments with the same sample, thus increasing data impact. In addition, even though SEC columns that allow for EVs separation from large volumes are finally emerging, they are still very costly and a few of them would be required in case of repeated usage. Our present UC/SEC combination takes advantage of the SEC impact on sample quality without the requirement of multiple columns in order to process such starting material. Alternatively, here we propose a rather cheap and sustainable method that consecutively has more potential for a wide use and would allow for a better standardization of techniques among teams. As the EVs community is in need of a general improvement of data specificity, we believe our easily accessible alternative could be of great help, especially to small research teams. 

For the same reasons, our method combination could also benefit the biomarker discovery in EVs [5]. For instance, better separation of EVs from freely circulating material, such as apoliproteins, in blood would allow for improved identification of EVs-specific biomarkers. As, for example, cancer-derived EVs are believed to travel very long distances to set up metastatic sites, improved plasma-derived EVs concentration could have highly sensitive clinical applications [5]. Nevertheless, as mentioned in the MISEV2018, definitive association of a biomarker with EVs might not be essential to such application. According to the authors, even if it just co-isolates with EVs, such biomarker is valuable as long as it can be associated to any clinical benefit (for diagnosis or prognosis for example) [2]. Yet, one could argue that better EVs separation leading to higher sample purity might provide more specific and thus more effective and stringent EVs-associated biomarkers. 

Despite that such a novel method can represent progress towards standardization, there is still room for improvement. For instance, while the decrease of particle concentration we could observe at the end of the protocol should be mostly due, in theory, to the actual purification process, it could also be due to material loss during the many handling, transfer and filtration steps. Furthermore, as we used only one GBM cell line for the present study, we have to acknowledge that our three method-protocol might present variable efficacy when performed with CM derived from cell lines or primary cells of different origins. Indeed, as we also observed in previous studies, EVs production and cargo are highly affected by their cell origin [5]. Nevertheless, even though an extended work would confirm such assumption, we believe that our present three method-protocol to be applicable to any sorts of biofluids, including CM from immortalized and primary cell lines. Furthermore, extended comparison of our present protocol to other available method combinations will be needed in the near future in order to fully assess its efficacy [14]. Finally, while the present protocol is optimized for the specific recovery of sEVs, which received most of the field attention over the last 10 years, interest in m/lEVs and larger particles is slowly growing [15]. As these EVs sub-populations might also be involved in key mechanisms in both normal and pathological conditions, there is a growing need for innovative methods for precise and reliable separation of these different EVs sub-populations. For instance, an additional SEC step could be added to the present protocol, following the 10,000× *g* UC step in order to separate m/lEVs from other membrane debris and contaminants. Such work would then be a highly valuable follow-up study to the present report.

## 3. Materials and Methods

### 3.1. Cells and Reagents

U118 glioblastoma (GBM) cells (ATCC) were maintained in Dulbecco’s Modified Eagle Medium (DMEM, Sigma-Aldrich, Gillingham, UK). Cell line culture medium was supplemented with 100 Units mL^−1^ penicillin, 100 µg mL^−1^ streptomycin, 2 mM L-glutamine (PSG, Sigma-Aldrich, Gillingham, UK) and 10% heat inactivated fetal bovine serum (FBS, Sigma-Aldrich, Gillingham, UK). 

### 3.2. Differential Ultracentrifugation for Extracellular Vesicle Concentration 

In order to collect sEVs derived from GBM cells, cells were seeded in 4 to 5 × 175 cm^2^ flasks and grown in 10% FCS medium until they reach confluence. Then, cells were washed with sterile PBS and 15 mL of corresponding serum free medium was added to each flask for 24 h. Following this incubation, conditioned medium (CM) was collected from each flask, pooled together in 2 × 50 mL falcon tubes and kept at either 4 °C for a very short time (up to 24 h) or at −20 °C for longer periods (up to 6 months) before sEV concentration. In accordance with the latest Minimal Information for Studies of Extracellular Vesicles (MISEV2018), cell count at time of collection was recorded and used to normalize the final sEV concentration (particles mL^−1^ per cell). Cell number and viability were measured using a Countess ™ cell counter (Thermo Fisher Scientific, Life Technologies, Paisley, UK) following mixing of the cell suspension with 0.4% Trypan blue. Only CMs harvested from cell culture with >90% viability were stored. 

**Figure 4:**
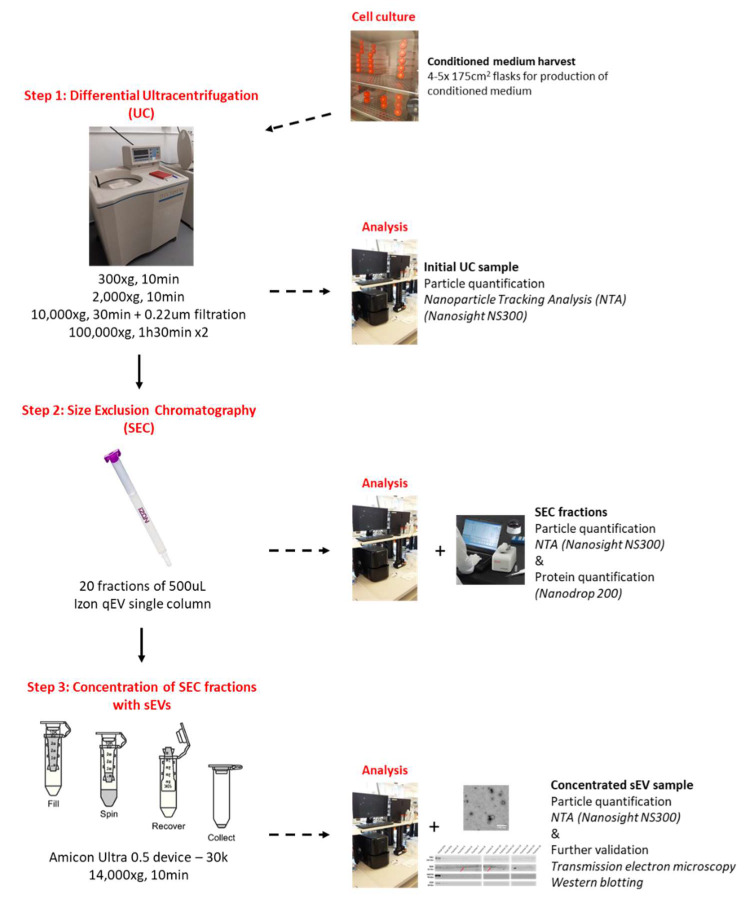
Three method-combination for concentrating EVs derived from cell culture medium. Cells are grown to confluence in 4 × 175 cm^2^ flasks to produce conditioned medium. Conditioned medium is then processed through the differential ultracentrifugation (UC) protocol in order to obtain an initial UC sample (step 1). The initial UC sample is then processed through a size exclusion chromatography column (SEC - Izon qEV single column) in the aim to separate putative EVs from protein contaminants (step 2). Following measurement of the particle and protein concentration, SEC fractions of interest are then pooled together and concentrated using Amicon ultra 0.5 devices (step 3). Final validation experiments confirm the sEVs concentration and the decreased protein contamination of the sample.

Concentration of sEVs was performed using an UC-based protocol [13]. Every step of the concentration protocol was performed at 4 °C. In total, 20 mL of stored CM was pipetted into each UC tube. An initial 300× *g* centrifugation was performed for 10 min to discard any floating cells from the CM, followed by a 10 min centrifugation step at 2000× *g* to remove any floating cell debris and dead cells (Hettich Universal 320R centrifuge, DJB Labcare Ltd., Newport Pagnell, UK). A 10,000× *g* UC step (Beckman optima LE 80-k ultracentrifuge, Beckman Type 70 Ti rotor, Beckman polypropylene centrifuge 14 × 89 mm tubes, full dynamic braking, **k_ad_**_j_ = 15,638, Beckman Coulter Ltd., High Wycombe, UK) was then performed for 30 min to remove any further cell debris and potential large vesicles (m/lEVs) from the CM. Finally, a first 100,000× *g* UC run was performed for 1 h 30 min to pellet the putative sEVs from the CM (Beckman optima LE 80-k ultracentrifuge, Beckman Type 70 Ti rotor, Beckman polypropylene centrifuge 14 × 89 mm tubes, full dynamic braking, k_adj_ = 494). Supernatant was stored at −20 °C. The UC pellet was then washed in filtered sterile PBS and centrifuged again for 1 h 30 min at 100,000× *g* in order to discard contaminants. The final pellet was re-suspended in 100 µL filtered sterile PBS and immediately characterized through nanoparticle tracking analysis (NTA). Protein concentration (µg/mL) of the final UC preparation was determined using a Nanodrop 200 spectrophotometer (Thermofisher Scientific, Life Technologies, Paisley, UK) (Figure 4).

### 3.3. Size Exclusion Chromatography for Extracellular Vesicle Separation from Protein Contaminants

Following the initial UC step, 20 µL of the original preparation (out of 100 µL) was kept at −20 °C for further analysis. The rest of the preparation (~80 µL) was diluted in filtered sterile PBS in order to reach a final volume of 150 µL. SEC was performed using qEV single size exclusion columns (separation size = 70 nm, iZON science, Oxford, UK). According to the manufacturer’s recommendations, the SEC column was first equilibrated using sterile PBS before the sample (150 µL) was loaded. As stated by the manufacturer, loading a 150 µL sample at the top of the column results in a 1 mL void volume and fractions of 500 µL. Following loading of the sample at the top of the column, fractions (20 in total) of 500 µL were immediately collected and kept on ice. First 7 fractions were then characterized through NTA. Protein concentration (µg/mL) of all fractions was determined using a Nanodrop 200 spectrophotometer (Thermofisher Scientific, Life Technologies, Paisley, UK). Fractions were then stored at −20 °C (Figure 4).

### 3.4. Concentration of SEC Fractions

SEC fractions with the highest concentrations of particles (as stated, based on NTA data) were concentrated in an Amicon Ultra 0.5 device – 30k (Merck milipore, Watford, UK). The centrifugal filter device was pre-rinsed with filtered PBS. Samples (500 µL at once) were loaded to the filter device and centrifuged at 14,000× *g* for 5–10 min at 4 °C. The putative concentrated sEVs preparation was characterized through NTA and was further processed or stored at −20 °C (Figure 4). 

### 3.5. Nanoparticles Tracking Analysis (NTA)

Vesicle concentration and size were determined using a Nanosight© NS300 and the Nanosight© NTA 3.2 software (Malvern Instruments, Malvern, UK). The following conditions were applied for the NTA analysis at the Nanosight instrument: temperature was 20–25 °C; viscosity was ~0.98cP; camera type was sCMOS; laser type was Blue488; camera levels were either 14 or 15; syringe Pump Speed was set to 70 AU; 5 measurements of 60 s each were recorded. Graphs show an average of at least 4 experiments. 

### 3.6. Coomassie Blue Staining

Samples were loaded, as stated, on 10% tris-glycine gels and run at 180 V and 40 mA for 100 min. The gels were then stained with Quick Coomassie Stain (Generon, Slough, UK) at room temperature overnight. Excess stain was removed through deionized water washes. Gels were viewed and captured by Criterion Stain Free Imager (Biorad, Watford, UK). 

### 3.7. Western Blotting

Characterization of the sEVs was performed through western blotting by measuring the expression of the EV membrane associated marker CD9 (mainly associated with light sEVs) and Fibronectin (mainly associated with dense sEVs), and EV cytosolic marker HSP70 [2,15]. Standard western blotting protocol was performed as described before [16]. For the EV marker analysis, comparable amount of sEVs (as stated) was loaded on the SDS gel. Primary antibodies: anti-BSA (Merck Millipore 07–248, 1/500 dilution, Merck-Millipore, Watford, UK), anti-CD-9 (System Biosciences EXOAB-CD9A-1, 1/10000 dilution), anti-Fibronectin (Abcam ab2413, 1/1000 dilution), anti-HSP-70 (System Biosciences EXOAB-HSP70A-1,1/10000 dilution, Cambridge Bioscience, Cambridge, UK). Secondary antibodies used: Polyclonal Goat Anti-Rabbit/Mouse Immunoglobulins/HRP (Dako P0447/8, 1/3000 dilution, Agilent, CA, USA) antibodies and Anti-Rabbit Immunoglobulins/HRP (ExoAb antibody Kit, System Biosciences EXO-AB-HRP, 1/3000 dilution, Cambridge Bioscience, Cambridge, UK). Chemiluminescence was observed using a UVP Chemstudio instrument (Analytik Jena, London, UK) and the Vision Works software. All experiments have been repeated at least 3 times. 

### 3.8. Transmission Electron Microscopy

Transmission electron microscopy (TEM) has been performed on putative sEVs preparation in order to visualize and assess/confirm the size range of the vesicles, as described before [13]. Samples were visualized using a JEOL JEM1400-Plus (120 kV, LaB6) microscope (JEOL Ltd., Welwyn Garden City, UK) equipped with a Gatan OneView 4K camera at × 20 k magnification. In total, 10–15 pictures per grid were taken. 

## 4. Conclusions

Overall, the present study establishes an easy and affordable method for sEVs separation that provides both improved sample purity and particles’ concentration. We believe that the present 3 method-combination protocol will have a great potential for future in vitro functional studies.

## References

[1] Margolis L., Sadovsky Y. (2019). The biology of extracellular vesicles: The known unknowns. PLoS Boil..

[2] Théry C., Witwer K.W., Aikawa E., Alcaraz M.J., Anderson J.D., Andriantsitohaina R., Antoniou A., Arab T., Archer F., Atkin-Smith G.K. (2018). Minimal information for studies of extracellular vesicles 2018 (MISEV2018): A position statement of the International Society for Extracellular Vesicles and update of the MISEV2014 guidelines. J. Extracell. Vesicles.

[3] Wendler F., Favicchio R., Simon T., Alifrangis C., Stebbing J., Giamas G. (2016). Extracellular vesicles swarm the cancer microenvironment: From tumor–stroma communication to drug intervention. Oncogene.

[4] Wendler F., Stamp G.W., Giamas G. (2016). Tumor–Stromal Cell Communication: Small Vesicles Signal Big Changes. Trends Cancer.

[5] Lane R., Simon T., Vintu M., Solkin B., Koch B., Stewart N., Benstead-Hume G., Pearl F.M., Critchley G., Stebbing J. (2019). Cell-derived extracellular vesicles can be used as a biomarker reservoir for glioblastoma tumor subtyping. Commun. Boil..

[6] Qin J., Xu Q. (2014). Functions and application of exosomes. Acta Pol. Pharm. - Drug Res..

[7] Simon T., Pinioti S., Schellenberger P., Rajeeve V., Wendler F., Cutillas P.R., King A., Stebbing J., Giamas G. (2018). Shedding of bevacizumab in tumour cells-derived extracellular vesicles as a new therapeutic escape mechanism in glioblastoma. Mol. Cancer.

[8] Gardiner C., Di Vizio L., Sahoo S., Théry C., Witwer K.W., Wauben M., Hill A.F. (2016). Techniques used for the isolation and characterization of extracellular vesicles: Results of a worldwide survey. J. Extracell. Vesicles.

[9] Helwa I., Cai J., Drewry M.D., Zimmerman A., Dinkins M.B., Khaled M.L., Seremwe M., Dismuke W.M., Bieberich E., Stamer W.D. (2017). A Comparative Study of Serum Exosome Isolation Using Differential Ultracentrifugation and Three Commercial Reagents. PLoS ONE.

[10] Takov K., Yellon D.M., Davidson S.M. (2018). Comparison of small extracellular vesicles isolated from plasma by ultracentrifugation or size-exclusion chromatography: Yield, purity and functional potential. J. Extracell. Vesicles.

[11] Lozano-Ramos I., Bancu I., Oliveira-Tercero A., Armengol M.P., Menezes-Neto A., Del Portillo H.A., Lauzurica-Valdemoros R., Borràs F.E. (2015). Size-exclusion chromatography-based enrichment of extracellular vesicles from urine samples. J. Extracell. Vesicles.

[12] Guerreiro E.M., Vestad B., Steffensen L.A., Aass H.C.D., Saeed M., Øvstebø R., Costea D.E., Galtung H.K., Søland T.M. (2018). Efficient extracellular vesicle isolation by combining cell media modifications, ultrafiltration, and size-exclusion chromatography. PLoS ONE.

[13] Théry C., Amigorena S., Raposo G., Clayton A. (2006). Isolation and Characterization of Exosomes from Cell Culture Supernatants and Biological Fluids. Curr. Protoc. Cell Boil..

[14] Kowal J., Arras G., Colombo M., Jouve M., Morath J.P., Primdal-Bengtson B., Dingli F., Loew D., Tkach M., Théry C. (2016). Proteomic comparison defines novel markers to characterize heterogeneous populations of extracellular vesicle subtypes. Proc. Natl. Acad. Sci. USA.

[15] Giamas G., Hirner H., Shoshiashvili L., Grothey A., Gessert S., Kuhl M., Henne-Bruns D., Vorgias C.E., Knippschild U. (2007). Phosphorylation of CK1δ: Identification of Ser370 as the major phosphorylation site targeted by PKA in vitro and in vivo. Biochem. J..

[16] Corso G., Mäger I., Lee Y., Görgens A., Bultema J., Giebel B., Wood M., Nordin J., El Andaloussi S. (2017). Reproducible and scalable purification of extracellular vesicles using combined bind-elute and size exclusion chromatography. Sci. Rep..

